# Cutting the ovarian surface improves the responsiveness to exogenous hormonal treatment in aged mice

**DOI:** 10.1002/rmb2.12345

**Published:** 2020-08-26

**Authors:** Takashi Umehara, Nao Urabe, Toshiki Obata, Takashi Yamaguchi, Atsushi Tanaka, Masayuki Shimada

**Affiliations:** ^1^ Graduate School of Integrated Sciences for Life Hiroshima University Higashi‐Hiroshima Japan; ^2^ Saint Mother Obstetrics and Gynecology Clinic Institute for ART Fukuoka Japan

**Keywords:** angiogenesis, control ovarian stimulation, cutting the ovarian surface, low responder, ovarian fibrosis

## Abstract

**Purpose:**

Ovarian vascular abnormality and ovarian fibrosis are observed in the low responder patients and aging mice. Vascularization and fibrosis are regulated by injury‐repair system, such as wound. Thus, in this study, the authors tried to investigate the effect of the surgical treatment to ovarian surface with cutting on the functions of ovary in aging mouse model, gc*Nrg1*KO.

**Method:**

The ovarian surface of gc*Nrg1*KO was surgically cut, and then the ovary was returned inside of bursa ovarica. To assess the effect of cutting on fertility, mating test, smear analysis, and exogenous hormonal treatment were done. Additionally, the histological analysis was used for observing the remodeling of ovarian stroma after the surgical approach.

**Result:**

Ovarian fibrosis disappeared at 7 days after surgery. With the abrogation of fibrosis, the blood vessels were fluently observed around the follicles, and the follicular development was re‐started. The responses against exogenous hormone were recovered at 21 days after the surgery, and estrous cycle and delivery were also recovered by the surgery and the fertility was maintained for 3 months.

**Conclusion:**

This cutting method of ovarian surface becomes a good option against low responder patients.

## Graphical abstract

Ovarian vascular abnormality and ovarian fibrosis are observed in the low responder patients and aging mice. Cutting of the ovarian surface induced tissue remodeling, angiogenesis and flexibility in fibrotic ovarian stroma. With these modifications, follicular development was re‐initiated to FSH‐responsible stages, and then the responsibility against exogenous hormonal treatment was recovered.

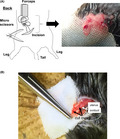

## 1 INTRODUCTION

Fertility decreases with increasing age, and natural pregnancy becomes difficult in most women over 40 years of age.^1^, ^2^ Infertility care using assisted reproductive technology (ART) with control ovarian stimulation (COS) supports women who desire pregnancy.^3^, ^4^ COS is an exogenous FSH/hMG treatment method to collect a large number of mature oocytes that are used for in vitro fertilization (IVF).^5^ However, the number of collected oocytes is limited in some patients who receive COS treatment, and these cases are called “low responders” or “poor responders.” Low responders are associated with increasing age, and the ratio of low responders increases 3‐fold in women over 40 years old compared with the ratio in women under 40 years old.^6^ Therefore, a low responder is thought to be the first step of ovarian aging.^7^, ^8^, ^9^ In low responders, a high level of FSH and a low level of anti‐Mullerian hormone (AMH) are commonly observed.^9^, ^10^, ^11^ AMH is produced in the granulosa cells of follicles from the primary stage to the antral stage, and the secretion level is higher in the secondary stage and antral stage, which are responsible for FSH, than in the primary stage.^12^ Thus, the decreased number of developing follicles, the decreased sensitivity to FSH in the follicles, and/or the insufficient supply of FSH to the follicles are considered the causes of low responders.

In our previous study, *Nrg1^flox/flox^*; *Cyp19‐Cre* mice (gc*Nrg1*KO) at 6 months of age showed similarities with low responders, such as the high circulating level of FSH, the low level of AMH, the abnormal estrous cycle and the decreased number of ovulated oocytes, even after exogenous hormonal treatment. Additionally, follicular development was arrested in the secondary follicles.^13^ Follicular development from the secondary to the antral stage is regulated by FSH secreted from the pituitary gland, indicating that the low sensitivity to FSH of secondary follicles and/or the insufficient supply of FSH by the deficient circulating system but not by limited secretion might induce follicular arrest in the secondary stage in the ovaries of gc*Nrg1*KO mice.^14^, ^15^, ^16^ Feng et al^17^ used the CLARITY approach and reported that the number of blood vessels was increased near the follicles during the developmental period from the secondary to antral follicle stages. Additionally, the disruption of *Vegfa* and the injection of a VEGF inhibitor dramatically decreased the number of ovulated oocytes during the hormone treatment cycle in mice.^17^ Interestingly, in the ovaries of low responders, blood flow in the ovarian stroma is decreased,^18^, ^19^ suggesting that insufficient supply of FSH by abnormal vessel formation in the ovary would be one of causes induced follicular arrest at the secondary stage in low responders.

Angiogenesis is strongly associated with injury and is activated as a “repair system” of tissue after injury.^20^ The first step after injury is that the expression of *Vegf* is dramatically increased; second, vascular epithelial cells are activated, and finally, blood vessels grow. Thus, we hypothesized that surgical cutting of the ovary could reactivate angiogenesis in the ovaries of low responders. In gc*Nrg1*KO mice, ovarian stromal fibrosis was observed to have similarities with that of low responders.^13^ It is well known that fibrosis is dependent on the surrounding mechano‐condition of cells.^21^ The cellular morphology of hepatocytes changes to that of fibrotic cells under rigid conditions where the cells are stretched by contact with the extracellular matrix (ECM) or cells to form stress fibers in the cells.^22^, ^23^ The formation of stress fibers is linked with the accumulation of collagen in cells,^24^ indicating that cutting would relieve cells of rigid conditions, and then could reduce stress fibers and collagen accumulation in ovarian stromal cells.

Therefore, in this study, we tried to surgically cut the surface of the ovary in a model of low responders, gc*Nrg1*KO mice, to recover ovarian function. Using small surgical scissors, the surface of ovaries was surgically cut and then returned inside the bursa ovarica. To assess the effect of ovarian cutting on fertility, a mating test, smear analysis, and hormonal treatment were performed. Additionally, histological analysis was used to observe the remodeling of the ovarian stroma after the surgical approach.

## 2 MATERIALS AND METHODS

### 2.1 Materials

Pregnant mare serum gonadotropin (PMSG/eCG) and hCG were purchased from Asuka Seiyaku. Routine chemicals and reagents were obtained from Sigma‐Aldrich or Nakarai Chemical Co.

### 2.2 Animals

Conditional depletion of *Nrg1* in ovarian granulosa cells (gc*Nrg1*KO) was achieved by crossing *Cyp19‐Cre* mice^25^ with *Nrg1* ^flox/flox^ mice.^26^ The mutant mouse strains were on a C57BL/6 background.

Animals were housed in the Experimental Animal Center at Hiroshima University under a 14‐hour light, 10‐hour dark schedule and provided with food and water ad libitum. Animals were treated in accordance with the NIH Guide for the Care and Use of Laboratory Animals, as approved by the Animal Care and Use Committee at Hiroshima University (30‐63, C18‐20‐2).

### 2.3 Cutting the ovarian surface

**FIGURE 1:**
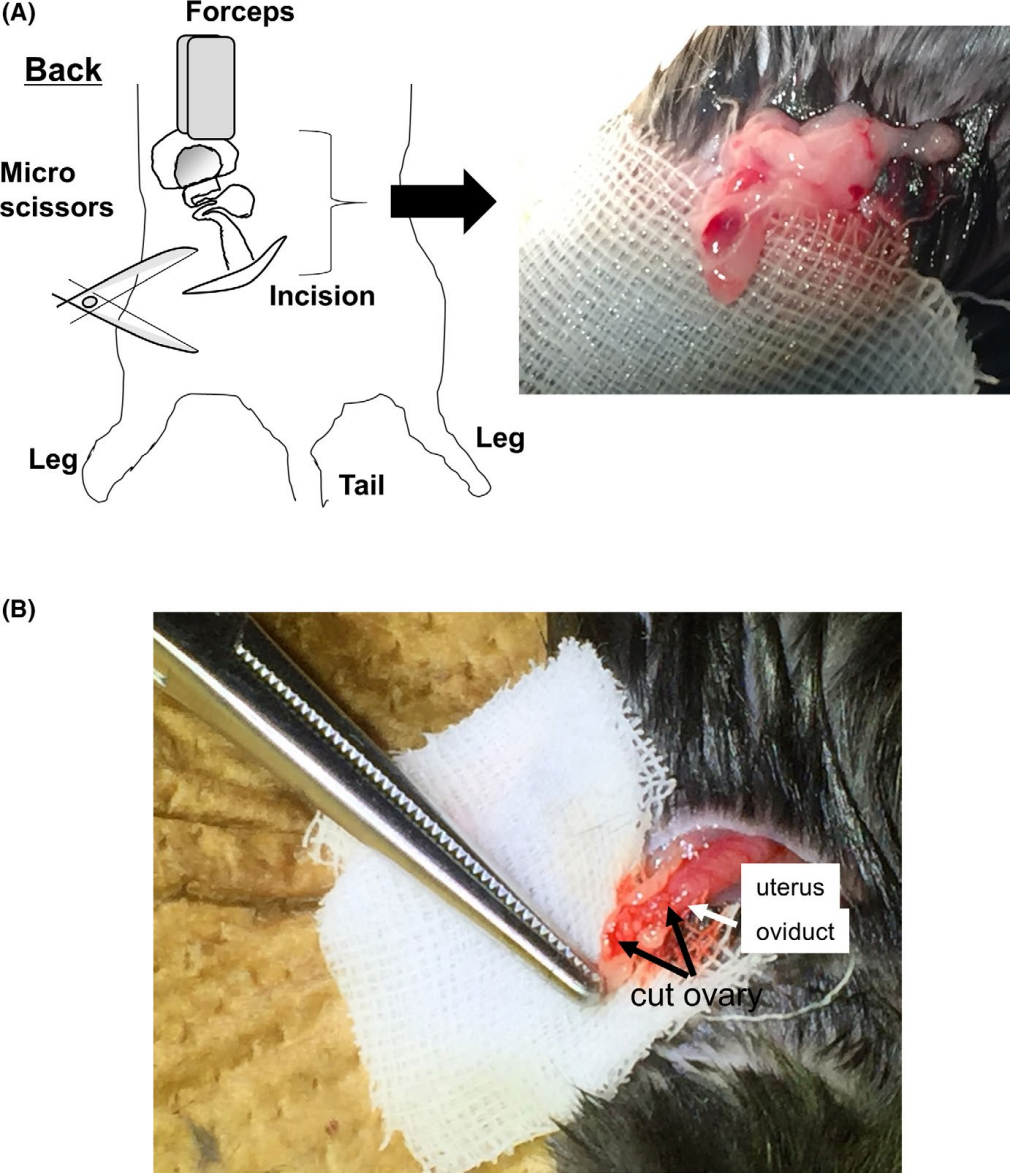
Overview of the cutting method of the ovary. Six‐month‐old gc*Nrg1*KO female mice were anesthetized, and then a small incision in the skin of the left back was made using scissors (A). The left ovary with the oviduct and uterus was pulled out onto gauze using forceps (B). Under the microscope, a small incision in the balsa was made. The surface of the ovary was cut with micro‐scissors 3 to 5 times being careful not to divide the ovary

An overview of the cutting of the ovarian surface is shown in Figure 1. Six‐month‐old gc*Nrg1*KO female mice were anesthetized using somnopentyl (Kyoritsu Seiyaku) and isoflurane (Pfizer Japan), and then a small incision in the skin of the left back was made using scissors (Figure 1A). The left ovary was pulled out on the gauze using forceps (Figure 1B). Under the microscope, a small incision in the balsa was made. The surface of the ovary was cut with micro‐scissors three to five times being careful not to divide the ovary. A video of this process is shown in Video S1. The ovaries were returned inside the body, and the skin was sutured after the operation. Cutting was also performed on the other side with the same method.

Four days after surgery, the ovaries were collected in order to assess tissue repair. Seven days after surgery, the ovaries were collected for histological analysis using PSR staining and for immunological analysis using IHC. Using the other gc*Nrg1*KO female mice after cutting the ovarian surface, the estrous cycle was analyzed 7 days after surgery, and then a mating test or superovulation treatment was performed.

### 2.4 Morphological analysis using hematoxylin and picrosirius red staining

The ovaries were fixed in 4% (w/v) paraformaldehyde/PBS overnight, dehydrated in 70% (v/v) ethanol, and embedded in paraffin. Paraffin‐embedded ovarian sections taken at intervals of 30 μm were mounted on slides. Routine hematoxylin and picrosirius red staining were performed for histological examination by light microscopy. Digital images were captured using a Keyence BZ‐9000 microscope (Keyence Co.). The collagen‐positive area was measured using 3 images per section with BZ‐II application software.

### 2.5 Immunohistochemistry

The ovaries were fixed in 4% (w/v) paraformaldehyde/PBS overnight, dehydrated in 70% (v/v) ethanol, and embedded in paraffin. The paraffin‐embedded fixed sections (4 μm) were deparaffinized in xylene washes and quenched with 3% hydrogen peroxide in methanol. The sections were incubated with 20% (v/v) nonimmune goat serum/PBS to block nonspecific sites, followed by incubation with primary antibodies overnight at 4°C (1:100 of anti‐FOXO1 antibody (catalog #2880; Cell Signaling) or 1:100 of anti‐CD31 antibody (catalog #ab28364; Abcam). The positive signals were visualized using a VECTASTAINE Life ABC rabbit IgG kit (Vector Laboratories) according to the manufacturer's recommendations. Digital images were captured using a Keyence BZ‐9000 microscope.

### 2.6 Vaginal smear analysis

Vaginal smear analyses were carried out according to Rascop et al.^27^ Smears were obtained daily between 09:00 and 10:00 am The fire‐polished, shortened tip of a Pasteur pipet was placed at the vagina.

### 2.7 Mating test

The mating experiment was conducted using three female mice in each group, (*Nrg1* ^flox/flox^ [WT], *Nrg1* ^flox/flox^;*Cyp19a1*‐*Cre* [gc*Nrg1*KO] and gc*Nrg1*KO, after ovarian cutting [cutting]). Adult WT male mice were placed in each cage for 3 months, and the number of pups in each litter and the days of pregnancy were recorded.

### 2.8 The collection of ovulated cumulus‐oocyte complexes (COCs) and in vitro fertilization

Twenty‐one days after surgery, the mice were injected intraperitoneally (ip) with 4 IU of eCG (Asuka Seiyaku) to stimulate preovulatory follicle development, followed by 5 IU of hCG (Asuka Seiyaku) 48 hours later to stimulate ovulation. In vitro fertilization was analyzed as described previously.^28^ COCs that were collected from the oviduct 16 hours after hCG administration were placed in 50 μL of human tubal fluid (HTF) medium. Spermatozoa were collected from the cauda epididymis of each mouse genotype into 500 μL of HTF medium. After 60 minutes of incubation to induce sperm capacitation, the spermatozoa were introduced into the fertilization medium at a final concentration of 1000 spermatozoa/μL. Twelve hours after insemination, all gametes were further cultured for an additional day in the developing medium (KSOM + AA; Millipore) to check for development to the blastocyst stage.

### 2.9 Statistics

Statistical analyses of the data from three or four replicates for comparison were carried out by either Student's *t* test or one‐way ANOVA followed by Student's *t* test (Statview; Abacus Concepts, Inc).

## 3 RESULTS

### 3.1 Ovarian shape recovers 4 days after cutting

**FIGURE 2:**
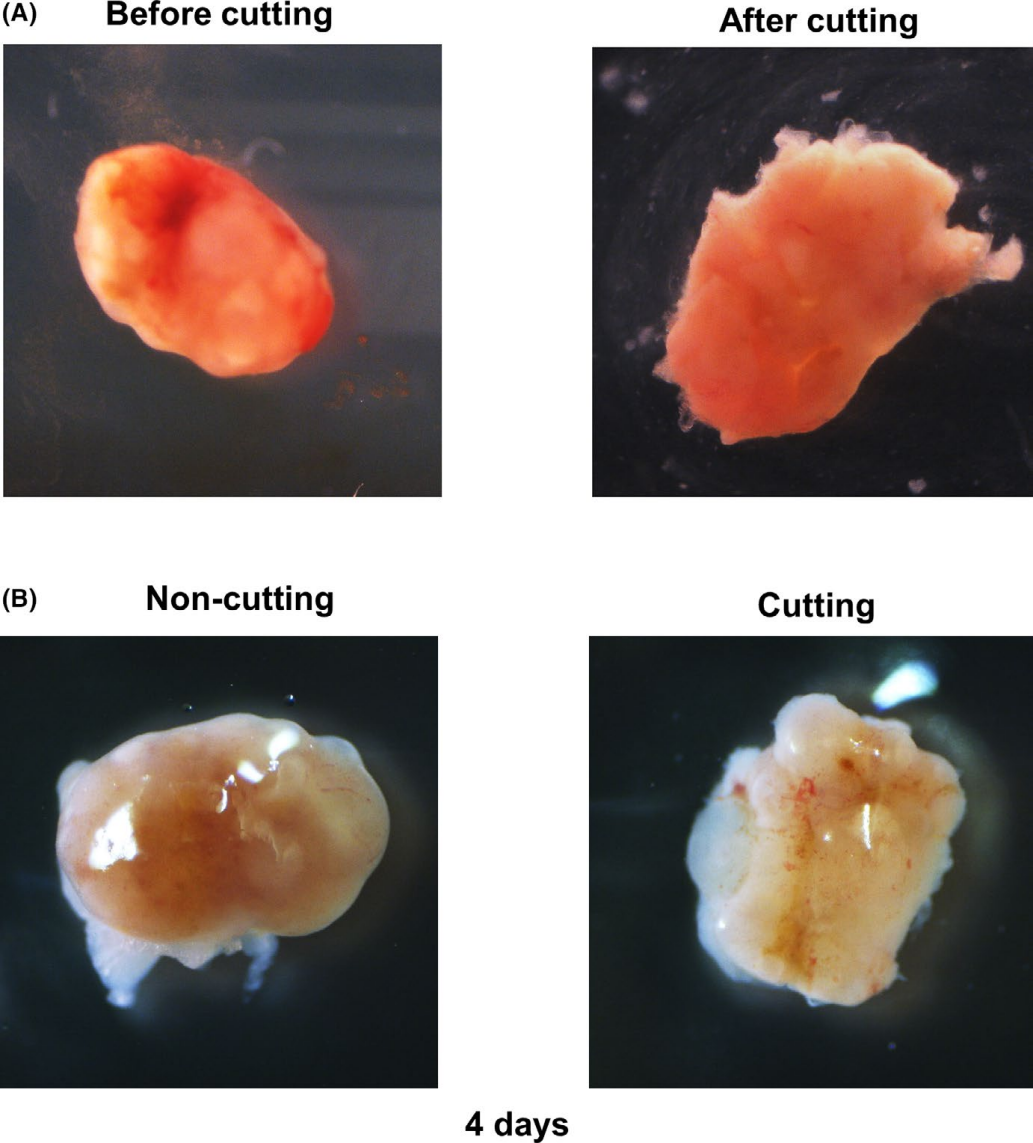
Ovary is repaired 4 d after cutting the ovarian surface. A, Image of the ovary before/after cutting. B, Image of the ovary 4 d after cutting

The pictures of the ovary after cutting are shown in Figure 2A. The ovaries were returned inside the body. Four days after the operation, the ovary was repaired, and some blood vessels were observed on the ovarian surface (Figure 2B).

### 3.2 Cutting of the ovarian surface abrogates ovarian fibrosis and induces vascular formation near the follicles

**FIGURE 3:**
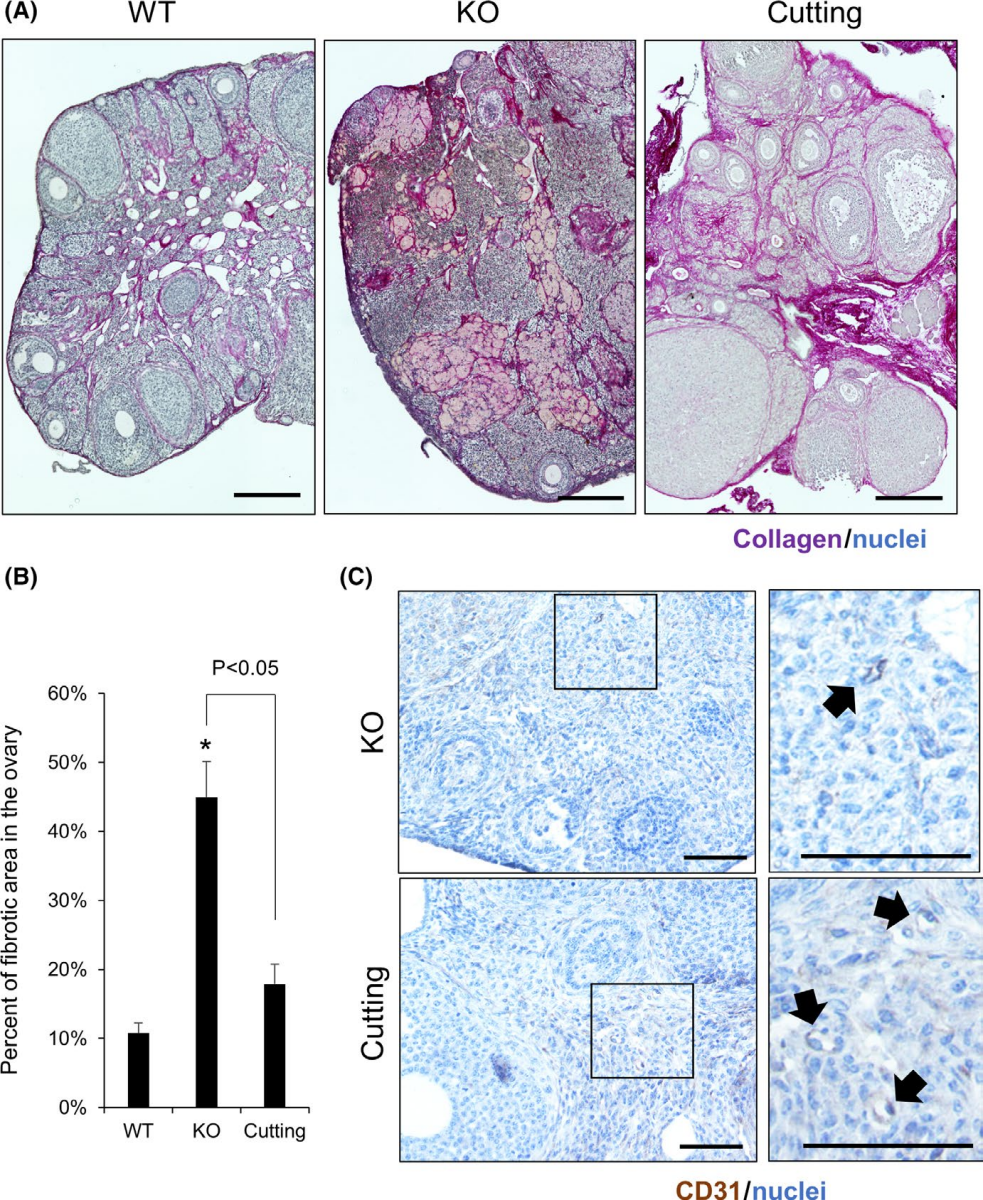
Ovarian fibrosis is abrogated by cutting the ovarian surface. A, PSR staining of the ovary in 6‐month‐old wild‐type (WT), gc*Nrg1*KO (KO), and gc*Nrg1*KO mice with cutting of the ovarian surface (cutting). Ovaries were collected 7 d after surgery and fixed using 4% paraformaldehyde. After embedding, 6‐μm sections were mounted on the slide. The slide was used for PSR staining. A deep purple signal indicated collagen. Scale bar indicates 200 μm. B, Percentage of fibrotic area in 6‐month‐old wild‐type (WT), gc*Nrg1*KO (KO), and gc*Nrg1*KO mice with cutting of the ovarian surface (cutting). The fibrotic area was measured using three images per section by BZ‐II application software. Asterisks (*) indicate significant differences compared with WT (*P* < .05). C, Immunohistochemistry of CD31, a marker of vascular endothelial cells, in the ovaries in gc*Nrg1*KO (KO) and gc*Nrg1*KO mice with cutting of the ovarian surface (cutting). Black arrows indicate blood vessels. Scale bar indicates 100 μm

Picrosirius red (PSR) staining was performed using the ovaries of wild‐type (WT), gc*Nrg1*KO, and gc*Nrg1*KO mice after cutting the ovarian surface. In the WT ovaries, multiple layers of secondary follicles, antral follicles, and the corpus luteum were observed. Additionally, thin layers of collagen were localized on the ovarian surface epithelium (OSE), the basal membrane of follicles, and the ovarian stromal area. In the ovaries of gc*Nrg1*KO mice, few follicles and corpus luteum were observed, and the cytoplasmic‐rich cells stained by PSR were occupied in the ovarian stromal area. Layers of collagen were observed in the OSE and stromal area similar to in WT ovaries; however, the layers were thickened compared with those of WT ovaries. Seven days after surgery, both follicles and the corpus luteum were fluently observed, and the cytoplasmic‐rich cells stained by PSR disappeared from the ovarian stroma (Figure 3A). To assess fibrosis in the ovary, the PSR‐positive area was measured in the ovary. The fibrotic area was significantly increased in gc*Nrg1*KO ovaries compared with WT ovaries (44.3 ± 5.8% vs 10.3 ± 2.3%). By cutting the ovarian surface, the area was dramatically decreased to <20%, and the percent was significantly lower than that in gc*Nrg1*KO ovaries (Figure 3B). With the decline in the fibrotic area in the ovarian stroma, blood vessels (arrows) were observed near the follicles after cutting, although blood vessels were rarely observed in the ovary before cutting (Figure 3C).

### 3.3 Cutting the ovarian surface enables ovulation using exogenous hormonal treatment in the model of low responders

**FIGURE 4:**
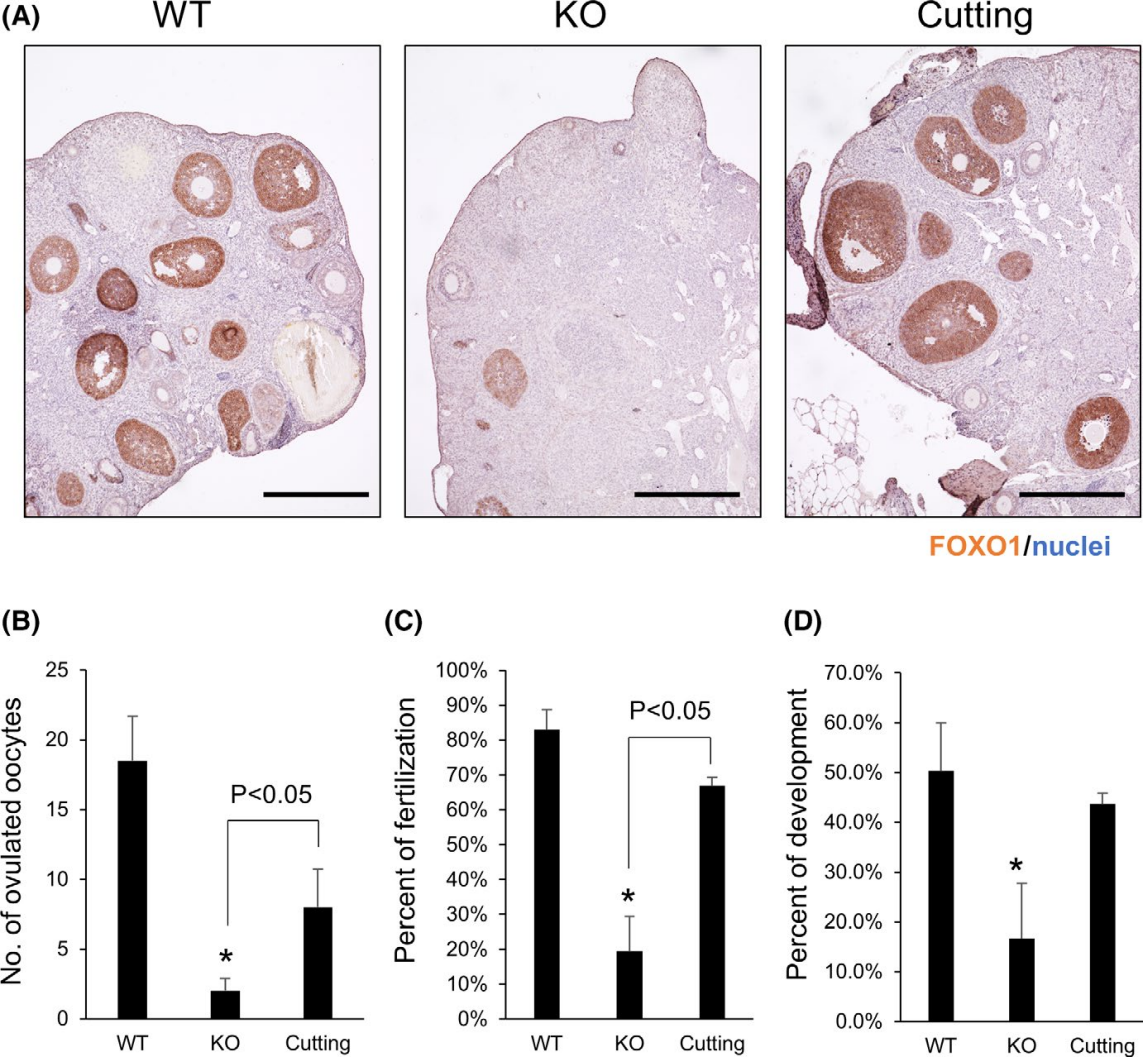
Responsibility against exogenous hormonal treatment is recovered by cutting the ovarian surface. A, Immunohistochemistry of FOXO1, a marker of follicular growth, in the ovaries of 6‐month‐old wild‐type (WT), gc*Nrg1*KO (KO), and gc*Nrg1*KO mice with cutting (cutting). The paraffin‐embedded sections were deparaffinized and used for immunohistochemical analysis using an anti‐FOXO1 antibody. Scale bar indicates 300 μm. B‐D, Ovulation number (B), the percent of fertilization (C), and the percent of development (D) after exogenous hormonal treatment using eCG and hCG 21 d after surgery. Sixteen hours after hCG injection, ovulated COCs were collected from the oviduct. COCs were inseminated with sperm in mHTF medium for 6 h, and then the oocytes were transferred to KSOM medium. The number of ovulated oocytes and 2‐cells was counted the next morning, and then the oocytes were cultured in KSOM medium for 5 d. The number of blastocysts was counted at 5 d

Immunohistochemical analysis was performed using an anti‐FOXO1 antibody, as FOXO1 is a marker of proliferating granulosa cells during the follicular development process. In the WT ovaries, several FOXO1‐positive follicles were observed, and the follicles were multilayer secondary follicles or antral follicles. However, FOXO1‐positive follicles were rarely observed in the gc*Nrg1*KO ovaries. Seven days after surgery, FOXO1‐positive follicles had increased, similar to in WT ovaries (Figure 4A). To check the responsibility against exogenous hormonal treatment, superovulation treatment using eCG and hCG was performed, the number of ovulated oocytes was counted, and then the oocytes were used for IVF. The results showed that 18.5 ± 3.1 oocytes were ovulated in WT ovaries; however, the number was significantly decreased in gc*Nrg1*KO ovaries (2.0 ± 0.8). Twenty‐one days after surgery, the number was significantly increased compared with that without cutting (KO vs cutting: 2.0 ± 0.8 vs 8.0 ± 2.7; Figure 4B). Additionally, the fertilization ratio was also significantly increased by cutting the ovarian surface (KO vs cutting: 19.4 ± 10.0% vs 66.9 ± 2.6%; Figure 4C). The developmental competence was slightly increased; however, significant differences were not observed (Figure 4D).

### 3.4 Cutting the ovarian surface restores fertility in the model of low responders

**FIGURE 5:**
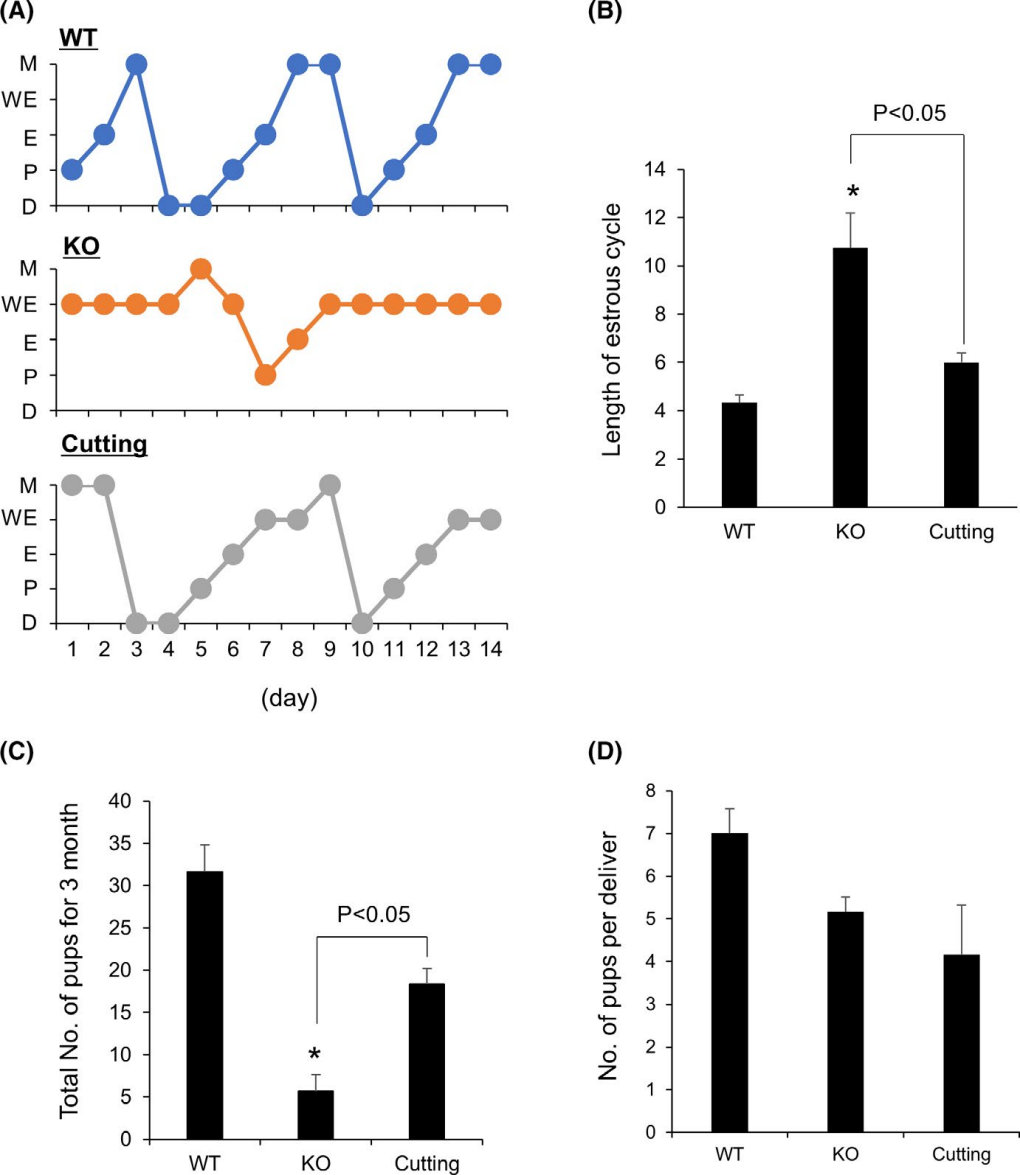
Cutting the ovarian surface restores fertility in the model of low responders. A, Estrous cycle of 6‐mo‐old wild‐type (WT), gc*Nrg1*KO (KO), and gc*Nrg1*KO mice with cutting of the ovarian surface (cutting) for 14 d. Seven days after surgery, smear analysis was performed for 14 d. B, The length of an estrous cycle. The days from diestrus to the next diestrus were calculated from the data smear analysis. Asterisks (*) indicate significant differences compared with WT (*P* < .05). C, D, The total number of pups for 3 months (C) and the number of pups per delivery (D) of 6‐month‐old wild‐type (WT), gc*Nrg1*KO (KO), and gc*Nrg1*KO mice with cutting (cutting). Females were mated with adult male mice 14 d after surgery for 3 mo. The number of pups was measured. Asterisks (*) indicate significant differences compared with WT (*P* < .05)

To understand the effect of cutting the ovarian surface on natural fertility, smear analysis was performed for 14 days from day 7 after surgery. In WT mice, proestrus, estrus, metestrus, and diestrus stages were observed, and an estrous cycle was 4‐5 days. However, in gc*Nrg1*KO mice, weak estrus, which had the characteristics of both estrus and metestrus, was observed, and the estrous cycle stopped at the weak estrus stage (Figure 5A). The length of the estrous cycle was significantly longer than that in WT mice (Figure 5B). Seven days after surgery, although weak estrus was observed on a few days, the length of an estrous cycle was significantly shorter than that of gc*Nrg1*KO mice (Figure 5A,B). These mice were mated with mature male mice for 3 months, and 32 pups were delivered by WT female mice. In gc*Nrg1*KO mice, the total number of pups was significantly lower than that in WT mice (approximately five pups); however, after cutting, the number recovered to 20 pups (Figure 5C). The number of pups per delivery was significantly lower in gc*Nrg1*KO mice than in WT mice. The number was not changed in gcNrg1KO mice with and without cutting (Figure 5D).

## 4 DISCUSSION

Fibrosis is observed in the low‐functioning ovaries of aged women.^29^ A similar phenomenon has also been reported in mice^30^ and primates^31^ with increasing age. Additionally, in our previous study, ovarian fibrosis was accelerated in gc*Nrg1*KO female mice with a low number of ovulated oocytes after exogenous hormonal treatment and with a decreased number of pups per delivery in the mating test.^13^ Therefore, ovarian fibrosis is associated with low fertility not only in aged women/females but also in low responders. In this study, the surgical treatment of ovaries with cutting recovered the responsibility against exogenous hormonal treatment with the abrogation of ovarian fibrosis.

It is well known that angiogenesis is less frequently observed in fibrotic tissue, such as liver, renal and lung tissues.^32^, ^33^ In this study, there were few blood vessels around the follicles in fibrotic ovaries; however, several blood vessels were observed around the follicles after ovarian cutting. Additionally, with an increasing number of blood vessels, ovarian fibrosis was abrogated. Generally, wounds enhance the inflammatory response at the injury point to recruit macrophages, which upregulate collagen expression for healing.^34^ However, the artificial wound did not induce fibrosis via the inflammatory response in the aged ovary. Similar to our cutting method, Kawamura et al^35^ reported that ovarian fragmentation induced the proliferation of granulosa cells in secondary follicles. In particular, ovarian fragmentation increased actin polymerization in the ovary. Actin remodeling, which is well known as the mechano‐reaction of cells, suppresses the activity of Hippo signaling.^36^ In the ovary, Hippo signaling is also disrupted by actin polymerization with ovarian fragmentation, and then the proliferation of granulosa cells is induced.^35^ In idiopathic pulmonary fibrosis, Hippo signaling is the inducer of fibrosis because the activation of Hippo signaling enhances the secretion of collagen.^37^ Zhao et al^38^ reported that the inactivation of Hippo signaling by melatonin decreased collagen expression, and idiopathic pulmonary fibrosis was recovered by this treatment. Additionally, Hippo signaling is associated with vascular epithelial cells, and vascular endothelial growth factor (VEGF) induces angiogenesis via inactivation of Hippo signaling.^39^ Thus, in our method, cutting the ovarian surface might induce depolymerization of actin to suppress Hippo signaling in ovarian stromal tissues, which might change the cell characteristics from fibrotic tissue to normal ovarian stroma. The cutting of the ovarian surface induces ovarian remodeling via mechano‐signaling to recover the functions of aged ovaries.

The relationship between the rigid condition surrounding follicles and follicular development has been analyzed using a follicular culture system of either alginate‐extracellular matrix gels, polyacrylamide gels, or fibrin‐alginate matrices. When 0.25%‐3% alginate gels were used for follicular culture,^40^, ^41^ the high stiffness condition of more than 1.5% alginate gels arrested follicular development at the secondary stage.^40^ Additionally, both follicular diameter and the expression of genes involved in follicular development were highest in follicles cultured using 0.25% alginate gels.^41^ Moreover, Shikanov et al (2009) used fibrin‐alginate gels with 5, 50, and 500 IU/mL of thrombin for follicular culture, in which the rigid condition was decreased with the concentration of thrombin. In their culture conditions, follicular survivability was the highest in the culture conditions using the gels with 50 IU/mL of thrombin.^42^ Therefore, the rigid condition around the follicle directly affects follicular development. In this study, our cutting method eliminated ovarian fibrosis in the ovarian stroma of the aging model. Collagen accumulated in the cytoplasm of fibrotic cells, where actin was polymerized in the ovarian stroma, indicating that ovarian hardening might be resolved by the cutting of the ovarian surface with the abrogation of ovarian fibrosis. Therefore, the modification of ovarian rigid conditions around follicles by ovarian cutting induces the re‐initiation of follicular development from the secondary stage.

FOXO (forkhead box O) is a well‐known transcriptional factor expressed in numerous types of cells.^43^ In the ovary, *Foxo1/3* double‐knockout mice in granulosa cells from the secondary to the antral stage showed infertility.^44^ Burns (2003) reported that FOXO1 was highly expressed in the granulosa cells of growing follicles from the secondary stage to the antral stage.^45^ Additionally, FOXO1 induced by FSH suppresses the premature luteinization of granulosa cells,^46^ indicating that FOXO1 is a marker of growing follicles where granulosa cells are activated by FSH. Therefore, the increasing number of FOXO1‐positive follicles indicates that ovarian cutting increases the number of growing follicles. Additionally, because the gene expression of *Fshr* was not difference between WT and our aging model (Figure S1), it was indicated that follicular growth was recovered by the improved supply of FSH but not the increased sensitivity to FSH after the ovarian cutting. In fact, with the increased number of FOXO1‐positive follicles, the responsibility against exogenous hormonal treatment, the estrous cycle, and natural fertility was recovered by ovarian cutting in gc*Nrg1*KO mice. Thus, cutting of the ovarian surface is a positive tool to recover the ovarian functions of low responders, although the method is an invasive surgical procedure.

Ovarian fibrosis is also observed in polycystic ovarian syndrome (PCOS).^47^, ^48^ One radical treatment is laparoscopic ovarian drilling (LOD) for PCOS patients.^49^ In the original LOD method, using an insulated unipolar needle electrode, three to eight punctures (each with a diameter of 3 mm and a depth of 2‐4 mm) are performed in the ovary by laparoscopy.^49^ Several papers showed that LOD recovered the ovulation rate and the pregnancy rate of PCOS patients.^50^, ^51^ Some studies have reported that the blood flow in the ovary or the production of steroid hormones derived from stromal cells is changed^52^, ^53^; however, the mechanisms by which ovarian functions are improved remain unclear. The localization of fibrosis is different between aged ovaries and PCOS ovaries; fibrosis is observed inside aged ovaries, while in PCOS ovaries, fibrosis is observed near the ovarian surface.^54^ Therefore, one possibility is that for aged ovaries in low responders, sectioning is required on the deep side to reach the ovarian stroma, but for PCOS, stimulation is sufficient near the surface area in the ovary to remove the fibrosis surrounding follicles.

In conclusion, cutting of the ovarian surface induced tissue remodeling, angiogenesis, and flexibility in fibrotic ovarian stroma. With these modifications, follicular development was re‐initiated to FSH‐responsible stages, and then the responsibility against exogenous hormonal treatment was recovered. Therefore, this cutting method of the ovarian surface may be a good option against patients who are low responders.

## CONFLICT OF INTEREST

MS received salary from Saint Mother Obstetrics and Gynecology Clinic as an adviser, and from Hiroshima Cryopreservation Service CO as a director. Other authors declare no conflict of interests.

Human rights: This article does not contain any studies with human patients performed by the any of the authors.

## ETHICAL APPROVAL

Animal studies were approved by the Animal Care and Use Committee at Hiroshima University (30‐63, C18‐20‐2).

## Supporting information

Fig S1

Video S1
