# Supplementary figures and images for: Cutting the ovarian surface improves the responsiveness to exogenous hormonal treatment in aged mice
Source: Reprod Med Biol. 2020 Aug 26;19(4):415–24. doi: 10.1002/rmb2.12345 (PMC7542011; doi:10.1002/rmb2.12345)

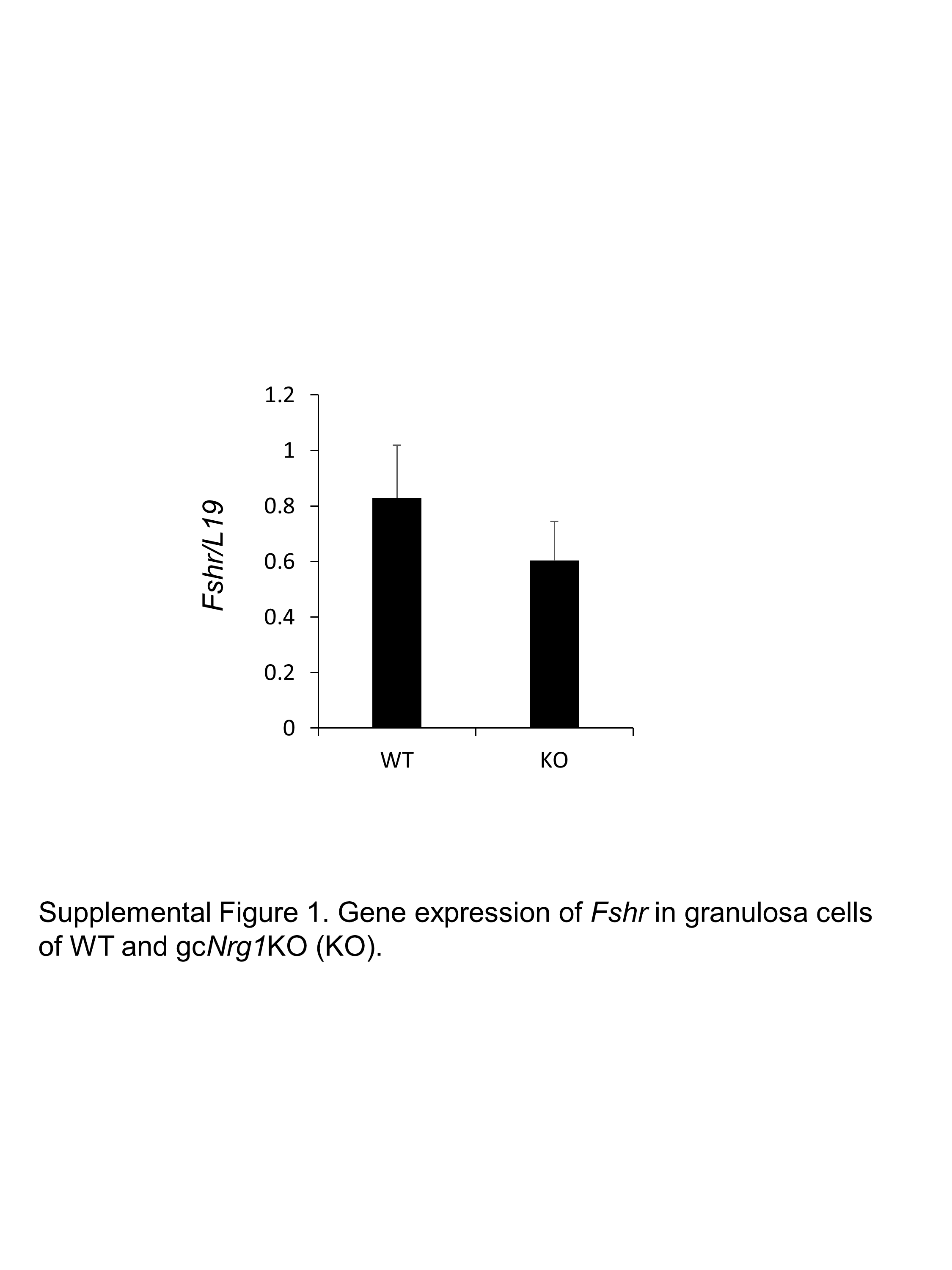

Supplement: Supplementary file 1 — Fig S1 [file RMB2-19-415-s001.TIF]
